# The Effect of Proximity‐To‐Failure on Perceptual Responses to Resistance Training

**DOI:** 10.1002/ejsc.12266

**Published:** 2025-02-17

**Authors:** Martin C. Refalo, Eric R. Helms, D. Lee Hamilton, Jackson J. Fyfe

**Affiliations:** ^1^ Institute for Physical Activity and Nutrition (IPAN) School of Exercise and Nutrition Sciences Deakin University Geelong Australia; ^2^ Sport Performance Research Institute New Zealand (SPRINZ) Auckland University of Technology Auckland New Zealand; ^3^ Florida Atlantic University Department of Exercise Science and Health Promotion Muscle Physiology Laboratory Boca Raton Florida USA

**Keywords:** discomfort, exertion, feelings, proximity‐to‐failure, repetitions‐in‐reserve, resistance training

## Abstract

Perceptual responses may influence how much pleasure or displeasure an individual experiences during or following resistance training (RT). Resistance‐trained males (*n* = 12) and females (*n* = 6) completed an 8‐week intervention involving two RT sessions per week. The lower limbs of each participant were randomised to perform the leg press and leg extension exercises either to (i) momentary muscular failure (FAIL) or (ii) a perceived 2‐RIR and 1‐RIR, respectively, for the entire intervention. In weeks one, four, and eight, post‐set ratings of perceived discomfort (RPD), and post‐session ratings of perceived exertion (RPE) and general feelings via feeling scale (FS) were measured. Data were analysed with Bayesian mixed‐effect models. When averaged over all time points measured, results showed slightly greater RPD for FAIL [5.1 (HDI: 4.2–6.0); *pd* = 100%] versus RIR [4.1 (HDI: 3.2–5.1); *pd* = 100%], greater RPE for FAIL [5.4 (HDI: 4.6–6.3); *pd* = 100%] versus RIR [4.3 (HDI: 3.5–5.1); *pd* = 100%], and more positive general feelings for RIR [1.2 (HDI: 0.7–1.8); *pd* = 100%] versus FAIL [0.3 (HDI: −0.3 to 0.8); *pd* = 86%]. Overall, assessing perceptual responses may help inform RIR prescription to promote desired outcomes whilst limiting negative feelings that may compromise long‐term adherence.

1

**Summary**

- Resistance training to momentary muscular failure (FAIL) results in slightly greater perceived discomfort, exertion, and worsened general feelings compared to terminating sets with 1‐ to 2‐repetitions in reserve (RIR), with the largest differences typically observed in week eight (albeit only slightly greater than weeks one and four).
- Although general feelings tended to worsen over time with FAIL, responses varied among individuals, with some reporting positive post‐exercise feelings and others negative. This variability highlights the importance of tailoring resistance training approaches to individual preferences and tolerances.
- Incorporating perceptual assessments during resistance training provides valuable feedback on individual experiences. Individuals who experience displeasure from reaching momentary muscular failure may benefit from terminating sets with 1‐ to 2‐RIR to minimise negative feelings, support comparable muscle hypertrophy and strength outcomes, and possibly enhance long‐term adherence.

## 2 Introduction

Resistance training (RT) promotes long‐term adaptations including skeletal muscle hypertrophy (Wackerhage et al. 2019) and strength development (Folland et al. 2007), as well as short‐term perceptual responses such as increased discomfort and exertion (M. C. Refalo et al. 2022). These perceptual responses may influence how much pleasure or displeasure an individual experiences during or following RT, possibly affecting their willingness to engage in long‐term RT (Rhodes et al. 2015). Although many RT variables may influence perceptual responses during and following RT (Fisher et al. 2017), the proximity to momentary muscular failure, quantified by the number of repetitions‐in‐reserve (RIR) remaining when a set is terminated, may be important (M. C. Refalo et al. 2023a). However, our previous scoping review (M. C. Refalo et al. 2022) only identified one study (Santos et al. 2019) investigating differences in perceptual responses between RT performed to varying proximities‐to‐failure. Considering adherence to RT is typically poor (Bennie et al. 2016, 2020) and the potential link between perceptual responses to exercise and long‐term adherence (Rhodes et al. 2015), further research investigating how proximity‐to‐failure influences perceptual responses is therefore warranted.

Although a previous study found higher perceived discomfort when RT sets were terminated at momentary muscular failure versus non‐failure (Santos et al. 2019), the specific RIR of the “non‐failure” group in this comparison was unclear, making it difficult to inform practical applications. As such, in our previous acute experimental trial (M. C. Refalo et al. 2023a) (published after the scoping review (M. C. Refalo et al. 2022)) participants rated their perceived discomfort, exertion and general feelings following three RT protocols performed to (i) momentary muscular failure (FAIL), (ii) a perceived 1‐RIR or (iii) a perceived 3‐RIR. It is worth noting that all perceptual responses worsened as proximity‐to‐failure neared (FAIL > 1‐RIR > 3‐RIR). However, it is unclear whether perceptual responses change with repeated exposure to RT over time. Exercise selection and number of exercises performed may also alter perceptual responses to RT. Indeed, existing data stems only from single‐exercise RT protocols (i.e., barbell bench press and smith machine squat) (M. C. Refalo et al. 2023a; Santos et al. 2019), which are likely not representative of real‐world RT programmes. Therefore, more research exploring the influence of proximity‐to‐failure on perceptual responses across an RT intervention is needed to inform RT prescription approaches.

### 2.1 Objectives

The primary objective of this study was to examine differences in perceived discomfort, exertion and general feelings between one, four, and 8 weeks of RT to either momentary muscular failure or with 1‐ to 2‐RIR in resistance‐trained individuals. In weeks one, four and eight we assessed ratings of perceived discomfort (RPD) after each set, and ratings of perceived exertion (RPE) and feeling scale (FS) ratings after each session. We employed Bayesian data analysis to (i) improve the precision of outcome estimates (by accounting for additional variables that may impact the results) and (ii) directly model uncertainty and intuitively present the results through posterior probabilities, allowing meaningful inferences about the influence of proximity‐to‐failure on perceptual responses (Kruschke et al. 2018).

## 3 Methods

### 3.1 Experimental Approach

**FIGURE 1:**
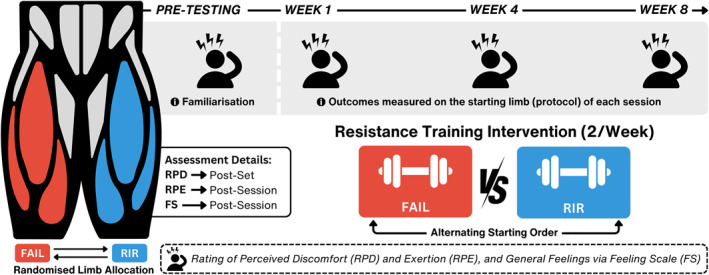
Schematic overview of study design and resistance training protocols. Participants completed 16 experimental sessions across the 8‐week intervention (two times per week separated by ∼72 h) with ratings of perceived discomfort (RPD), exertion (RPE) and general feelings via feeling scale (FS) assessed in weeks one, four and eight. The order of the starting limb (FAIL or RIR protocol) was randomised and alternated each session. Outcomes were only measured on the starting limb of each session. *RIR, repetitions‐in‐reserve.*

Data were obtained as part of a previously published study (M. C. Refalo et al. 2024). Resistance‐trained males and females completed an 8‐week RT intervention, with each lower limb randomised to perform RT either to (i) momentary muscular failure (FAIL) or (ii) a perceived 2‐RIR and 1‐RIR (RIR). Exercises performed were the unilateral leg press and leg extension. RT sessions were completed twice per week and separated by ∼72 h (Figure 1). The order of the starting limb (FAIL or RIR protocol) was randomised and alternated each session. For the first protocol (FAIL or RIR) completed by each participant in weeks one, four and eight, RPD was measured after each set (for both the leg press and leg extension), and both RPE and FS were measured after completion of the whole protocol.

### 3.2 Participants

Baseline participant characteristics are presented in Table 1. Male (*n* = 12) and female (*n* = 7) participants were (i) 18–40 years old, (ii) had no existing musculoskeletal injuries or neuromuscular disorders, (iii) confirmed they had not used anabolic steroids or illegal agents known to increase muscle size for the previous year, and (iv) had at least three years of RT experience (with three or more RT sessions per week) (Santos Junior et al. 2021). Of the 18 participants, 16 (89%) had worked with a personal trainer face‐to‐face and nine (50%) previously competed in powerlifting or bodybuilding.

**TABLE 1:** Baseline participant characteristics. An overview of the relevant characteristics for each participant included in data analysis. Shown below is the highest quadriceps set volume completed by participants, which was increased by 20% halfway throughout the intervention. kg, kilogrammes; p/w, per week; y, years.

	Males (*n* = 12)		Females (*n* = 6)	
Variable	Mean ± *SD*	Range	Mean ± SD	Range
Age (y)	26.9 ± 3.1	20–31	30.0 ± 5.8	24–38
Bodyweight (kg)	82.6 ± 6.0	75–94	62.8 ± 5.4	57–72
RT experience (y)	7.8 ± 2.6	4–13	7.5 ± 2.3	5–10
RT frequency (p/w)	4.8 ± 0.9	3–6	4.7 ± 0.8	4–6
Quadriceps set volume	12 ± 1	10–14	14 ± 2	12–17

#### 3.2.1 Sample Size Justification

This was a secondary analysis of another study (M. C. Refalo et al. 2024). The target sample size of that main study (20 participants) was based on the following pragmatic considerations: (i) recruiting more than 20 participants was not feasible due to time and associated costs to complete data collection and analysis and (ii) this sample size was greater than similar within‐subject unilateral pre‐post studies investigating the influence of RT proximity‐to‐failure on muscle hypertrophy (Santanielo et al. 2020; Andersen et al. 2021; Lacerda et al. 2020). Initial sample size calculations were performed (M. C. Refalo et al. 2024), with the final decision to employ Bayesian statistical methods to generate posterior distributions to (i) account for uncertainty in parameter estimates where limited data are available, and (ii) provide a full probability distribution to interpret not only the point estimates (or single effect sizes) but also the entire range of plausible values (Kruschke et al. 2018).

### 3.3 Procedures

The following protocols were part of the main study described elsewhere (M. C. Refalo et al. 2024).

#### 3.3.1 Exercise Control

Participants were asked to not perform (i) high‐intensity aerobic exercise during the intervention, and specifically, (ii) any lower‐body RT or aerobic exercise 24‐h before each study visit. Participants could perform additional moderate‐intensity RT involving muscle groups other than the quadriceps, but exercise constraints were employed to minimise potential confounding influences (described in Supplementary Table 1.1).

#### 3.3.2 Menstrual Cycle Considerations

Considering that all female participants completed both unilateral protocols, thus acting as their own “controls”, and that recent meta‐analyses indicate that both (i) menstrual cycle phase (McNulty et al. 2020) and (ii) modern oral contraceptive use (Elliott‐Sale et al. 2020) have the most trivial effects on exercise performance at the group level, females commenced the intervention period at any timepoint throughout their menstrual cycle and no timing considerations were made for post‐testing. If participants experienced menstrual symptoms during the study period that they believed affected RT performance, study visits were rescheduled as necessary.

#### 3.3.3 Pre‐Testing Sessions

##### 3.3.3.1 Exercise Technique

Leg press and leg extension exercises were performed with standardised technique as described previously (M. C. Refalo et al. 2024). Participants were instructed to perform the concentric (lifting) phase of each repetition as fast as possible, followed by a controlled eccentric (lowering) phase (∼2 s). See Supplementary Figure 1.2 for images of equipment and demonstration of exercise technique.

##### 3.3.3.2 Repetition‐Maximum Load Assessment

To determine starting loads, participants completed four repetition‐maximum (RM) assessments (8–10‐RM for the leg press and 10–12‐RM for the leg extension per limb) in pre‐visit one. An overview of the procedures and participant instructions can be found elsewhere (M. C. Refalo et al. 2024). Testing was repeated until the participant reached momentary muscular failure on the 9th, 10th or 11th repetition. Once the 8–10‐RM load was established on the leg press, the process was repeated to determine leg extension 10–12‐RM loads (for both limbs). An experienced supervisor ensured safety and provided strong verbal encouragement for maximum lifting velocity.

##### 3.3.3.3 Repetitions‐To‐Failure Assessment

In pre‐visit two, participants completed two sets to momentary muscular failure with the loads determined in pre‐visit one for the leg press and leg extension. An overview of the procedures and participant instructions can be found elsewhere (M. Refalo et al. 2023b). All procedures were performed on both limbs, in a randomised manner. For familiarisation purposes, participants were asked to rate their perceived discomfort after each set performed, and their perceived exertion and general feelings after the completion of the session.

#### 3.3.4 Resistance Training Intervention

Participants performed both exercises unilaterally on both lower limbs twice per week for 8 weeks (Figure 1), with each limb randomly assigned to perform either the FAIL or RIR protocol. FAIL performed all sets to momentary muscular failure, whereas RIR performed leg press to 2‐RIR and leg extension to 1‐RIR, as described elsewhere (M. C. Refalo et al. 2024). To explore individual responses and increase the precision of RT effects on muscle hypertrophy (Scarpelli et al. 2022), set volume for each participant was equal to the weekly number of quadriceps sets they reported performing most recently and was equally distributed between the leg press and leg extension. Where a participant was performing ≥ 15 sets, a 20% decrease in volume was implemented (e.g., 16 sets—20% = 13 sets) to mitigate potential injury risk, excessive fatigue and prolonged session durations. Halfway through the intervention (at week five), participants increased set volume by 20%.

##### 3.3.4.1 Resistance Training Protocol

Participants commenced the first RT session on a random limb, with starting limbs alternated each session. Both exercises were completed before training the alternate limb. Four warm‐up sets were performed on the leg press, starting with the minimum load, working up to 50%, 65% and 85% of the 8–10‐RM load (for ten, eight, six and four repetitions, with two‐minute inter‐set rest periods). Two warm‐up sets were performed on the leg extension (50% and 65% of the 10–12‐RM load for five repetitions). Participants then performed their specified number of sets on each exercise with their individualised load. For both protocols, if the participants performed more repetitions than the RM load range, the load was adjusted on the subsequent set by 2.5–5 kg on the leg press and 1.25–2.5 kg on the leg extension. Four minutes rest was given between working sets on the leg press, 2 min for the leg extension and 5 min between exercises. After completing the RT protocol for the starting limb, a five‐minute rest period was provided before beginning the protocol on the opposite limb. If a participant experienced musculoskeletal discomfort that prevented them from performing either exercise, specific protocol modifications were made (described elsewhere (M. C. Refalo et al. 2024)). To ensure recovery and minimise residual fatigue, ∼72 h was allocated between RT sessions; however, 48–96 h were allowed for scheduling flexibility if participants were unable to schedule 72 h between sessions. All RT sessions were monitored by a qualified exercise professional (MR) and strong verbal encouragement was provided during each working set. Participants that completed 87.5% of scheduled sessions (14 out of 16 RT sessions) were included in the final analysis.

### 3.4 Outcome Measures

Outcome measures were recorded only from the starting limb of each RT session (alternated each session) to prevent ratings from being influenced by the residual effects of having trained the other limb.

#### 3.4.1 Perceived Discomfort

Immediately after completing each set, only for the starting limb in weeks one, four and eight, participants provided their RPD (Fisher et al. 2017). Participants were asked*: “how much discomfort did you feel in that set?”* and to rate their perceived discomfort during the set on a 1‐10 scale, whereby zero represents “no discomfort” and 10 represents “maximal discomfort”. During the initial repetitions‐to‐failure assessment (2.3.3.3), participants were comprehensively instructed as follows based on research by Fisher et al. (2017): “The scale begins at 0, which is described as no perceived discomfort. This can be likened to a perception of discomfort at a time where you feel no noticeable sensations relating to physical activity. The scale ends at 10, which is described as the maximum perceivable discomfort. This can be likened to a perception of discomfort where you could not imagine the sensations relating to physical activity being any more intense.”

#### 3.4.2 Perceived Exertion and General Feelings

Participants provided session‐RPE with the modified category ratio (CR‐10) scale (Foster et al. 2001; Sweet et al. 2004) and general feeling scores on the FS (Hardy et al. 1989). Ratings were recorded only for the starting limb within 5 minutes after completing the RT protocol (both leg press and leg extension) in weeks one, four and eight. These ratings reflected perceived exertion and general feelings for the entire RT protocol, whether FAIL or RIR. For both scales, participants were provided with standardised instructions on how to use the scales during the initial repetitions‐to‐failure assessment (Section 2.3.3.3). Participants were asked “*how hard was your workout*?” and to rate their perceived exertion for the session (up to that point) on a 0–10 scale. A rating of 0 represented complete rest with “no effort”, whereas a rating of 10 indicated “maximal effort”, corresponding to the most challenging RT session they had ever experienced (Sweet et al. 2004). Participants were also instructed to evaluate the entire RT session (up that point) when providing their response and it was made clear that the session‐RPE score was not representative of any RIR value (e.g., 8‐RPE did not represent 2‐RIR). Participants were also asked *“how do you currently feel?”* and to assess their general feelings towards the session (up to that point) with the FS, ranging from “+5”, which refers to “very good”, to “−5”, which refers to “very bad” (Hardy et al. 1989).

### 3.5 Statistical Analysis

To provide a flexible modelling approach and intuitive interpretation by reporting probabilities (Sweet et al. 2004), we analysed data with Bayesian linear mixed‐effect models using the “brms” (Bürkner, 2023) package in R (v 4.0.2; R Core Team, https://www.r‐project.org/). Considering the ordinal outcomes assessed in this study (i.e., RPE, RPD and FS) serve as proxies for underlying continuous constructs, we chose to treat ordinal measures as continuous to simplify interpretation and provide an estimate of the general relationship between predictors and the outcome. Posterior draws were extracted using “tidybayes” (Kay, 2023), estimated marginal effects were calculated using “emmeans” (Lenth, 2023), and the probability (i.e., percentage value ranging from 0% to 100%) that an estimate was in favour of a given protocol (FAIL or RIR) was calculated manually by examining the proportion of posterior draws that met the criteria of interest (e.g., > 0) and denoted as the probability of direction (*pd*). Models were generated to assess differences in outcome measures (i.e., RPD, RPE and FS) between protocols and explore the differences at three timepoints and for each exercise (where possible). Further model details, population‐level effects and final group‐level slope structures are displayed in Supplementary Table 2.1. Non‐informative priors (i.e., default “brms” priors) were used for all model parameters for all outcomes. All inferences were made from posterior samples generated using the Hamiltonian Markov Chain Monte Carlo method and via high‐density credible intervals (HDI). For each outcome, differences between protocols of 0.5–0.9 points were considered as ‘slight’ (if *pd* = 100%), whereas differences between protocols of ≥ 1 point were considered as ‘meaningful’ for each outcome. All raw data (in text and figures) are presented as mean and standard deviation. A comprehensive overview of the statistical analysis along with the R code used can be found on the Open Science Framework (https://osf.io/34d92/).

## 4 Results

### 4.1 Intervention Adherence

Mean participant adherence (i.e., percentage of completed sessions) was 97.5% (87.5%–100%). In some instances, sessions were completed over 11 weeks instead of 10 due to scheduling constraints. To maintain adherence, minor protocol modifications were made if a participant experienced musculoskeletal discomfort (e.g., muscular strains or knee joint pain) but was able to continue the study as mutually decided by the participant and the supervisor. Eight participants experienced minor musculoskeletal discomfort (FAIL = 5, RIR = 3), with two of the eight unable to perform the leg press in some weeks (in which case the remaining set volume was allocated to the leg extension). One participant experienced a muscular strain (limb = RIR) in the second week and had to cease participation, but once recovered (∼12‐week), re‐commenced the study from the start. All participants completed the study.

### 4.2 Repetitions‐In‐Reserve Prediction Accuracy

Summary of RIR accuracy can be found elsewhere (M. C. Refalo et al. 2024). Participants had a high absolute RIR accuracy; on average less than one repetition from the 1‐ and 3‐RIR targets on both exercises (Supplementary Table 3.4).

### 4.3 Resistance Training Variables

All resistance training variables recorded can be found elsewhere (M. C. Refalo et al. 2024) or in Supplementary Table 3.5. Average volume load and repetition volume across all sessions of the RT intervention were similar between FAIL and RIR.

### 4.4 Rating of Perceived Discomfort

**FIGURE 2:**
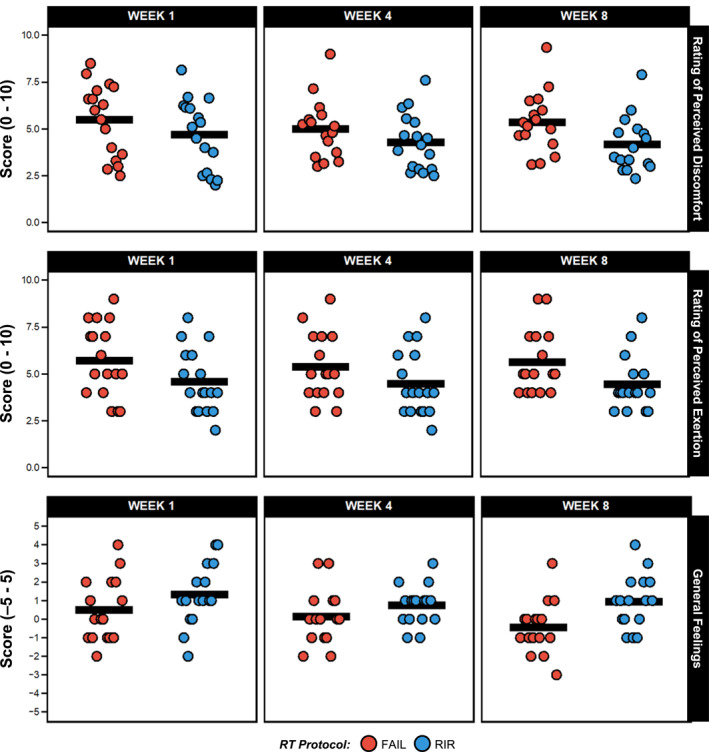
Raw rating of perceived discomfort and exertion, and general feelings via feeling scale in weeks one, four and eight for both FAIL and RIR. Data shown are raw values presented as both protocol means (with individual values), and the *SD* of protocol means can be found in Supplementary Tables 3.1 to 3.3. Data for rating of perceived discomfort is averaged over both the leg press and leg extension exercises.

**FIGURE 3:**
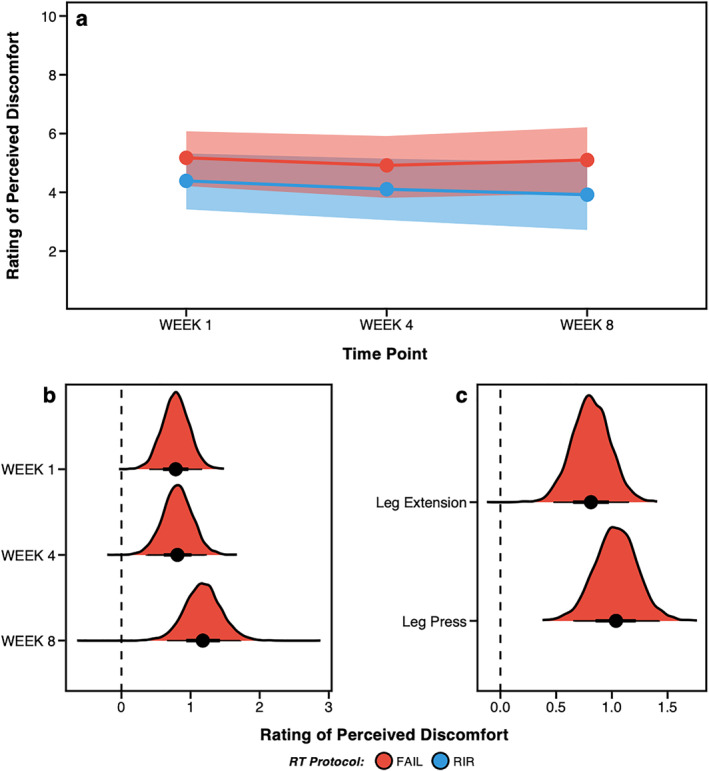
Bayesian estimates of rating of perceived discomfort (RPD) for FAIL and RIR in weeks one, four and eight (a) and posterior distributions of between‐protocol differences for each week (b) and exercise (c). Figure B/C displays the posterior distributions for between‐protocol differences along with the central tendency (i.e., point estimate = mean) and highest density credible intervals. Positive values in Figure B/C favour greater RPD for FAIL versus RIR.

Raw RPD in weeks one, four and eight are displayed in Figure 2 and Supplementary Table 3.1 and Bayesian estimates are shown in Figure 3A. When averaged across each exercise and timepoint measured, greater RPD was estimated for FAIL [5.1 (HDI: 4.2–6.0); *pd* = 100%] versus RIR [4.1 (HDI: 3.2–5.1); *pd* = 100%]. Slightly greater RPD was also estimated for FAIL [5.2 (HDI: 4.2–6.1); *pd* = 100%] versus RIR [4.4 (HDI: 3.4–5.3); *pd* = 100%] in week 1, for FAIL [4.9 (HDI: 3.8–5.9); *pd* = 100%] versus RIR [4.1 (HDI: 3.1–5.2); *pd* = 100%] in week 4 and for FAIL [5.1 (HDI: 4.0–6.2); *pd* = 100%] versus RIR [3.9 (HDI: 2.7–5.0); *pd* = 100%] in week 8. When averaged across each timepoint measured, greater RPD was also estimated for FAIL [5.3 (HDI: 4.2–6.2); *pd* = 100%] versus RIR [4.2 (HDI: 3.3–4.2); *pd* = 100%] when the leg press was performed and slightly greater for FAIL [4.7 (HDI: 3.8–5.8); *pd* = 100%] versus RIR [4.1 (HDI: 3.1–5.1); *pd* = 100%] when the leg extension was performed. Estimates for between‐protocol differences are shown in Table 2 and posterior distributions in Figure 3B/C.

**TABLE 2:** Estimates of between‐protocol differences (i.e., contrast between FAIL and RIR). Negative estimate values favour RIR and positive estimate values favour FAIL. Overall results are averaged over exercise and time. *pd, probability of direction.*

Outcome variable	Estimate (between‐protocol)	HDI	*pd*
Rating of perceived discomfort			
Overall	0.9	0.6 to 1.2	100%
Week 1	0.8	0.4 to 1.2	100%
Week 4	0.8	0.4 to 1.2	100%
Week 8	1.2	0.7 to 1.7	100%
Leg press	1.0	0.7 to 1.4	100%
Leg extension	0.8	0.5 to 1.2	100%
Rating of perceived exertion			
Overall	1.1	0.6 to 1.6	100%
Week 1	1.0	0.4 to 1.8	100%
Week 4	0.9	0.2 to 1.7	99%
Week 8	1.3	0.5 to 2.1	100%
General feelings (via feeling scale)			
Overall	−1.0	−1.5 to −0.4	100%
Week 1	−0.8	−1.6 to 0.1	96%
Week 4	−0.6	−1.6 to 0.3	91%
Week 8	−1.5	−2.4 to −0.6	100%

### 4.5 Rating of Perceived Exertion

**FIGURE 4:**
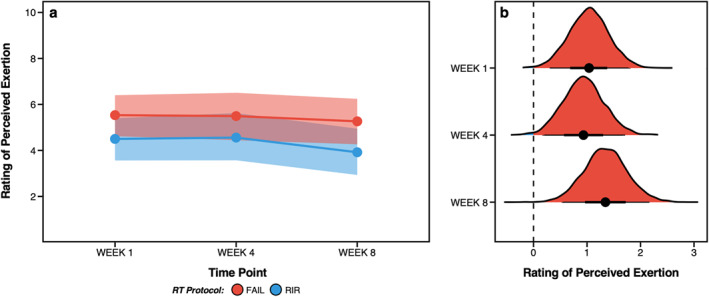
Bayesian estimates of rating of perceived exertion (session‐RPE) for FAIL and RIR in weeks one, four and eight (a) and posterior distributions of between‐protocol differences for each week (b). Figure B displays the posterior distributions for between‐protocol differences along with the central tendency (i.e., point estimate = mean) and highest density credible intervals. Positive values in Figure B favour greater session‐RPE for FAIL versus RIR.

Raw session‐RPE in weeks one, four and eight are displayed in Figure 2 and Supplementary Table 3.2 and Bayesian estimates in Figure 4A. When averaged across each timepoint measured, greater session‐RPE was estimated for FAIL [5.4 (HDI: 4.6–6.3); *pd* = 100%] versus RIR [4.3 (HDI: 3.5–5.1); *pd* = 100%]. Greater session‐RPE was also estimated for FAIL [5.5 (HDI: 4.6–6.4); *pd* = 100%] versus RIR [4.5 (HDI: 3.6–5.4); *pd* = 100%] in week 1, for FAIL [5.5 (HDI: 4.4–6.5); *pd* = 100%] versus RIR [4.6 (HDI: 3.6–5.6); *pd* = 100%] in week 4 (slight) and for FAIL [5.3 (HDI: 4.3–6.2); *pd* = 100%] versus RIR [4.0 (HDI: 2.9–5.0); *pd* = 100%] in week 8. Estimates for between‐protocol differences are shown in Table 2 and posterior distributions in Figure 4B.

### 4.6 General Feelings via Feeling Scale

**FIGURE 5:**
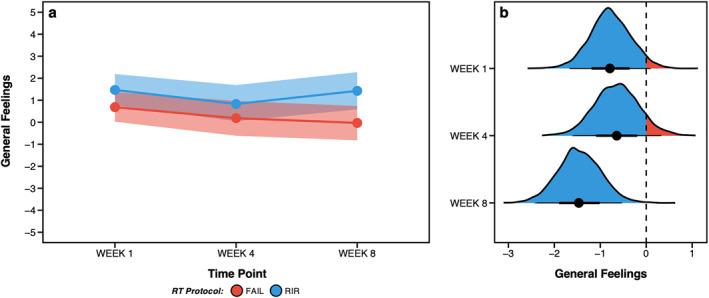
Bayesian estimates of general feelings via the feeling scale (FS) for FAIL and RIR in weeks one, four and eight (a) and posterior distributions of between‐protocol differences for each week (b). Figure B displays the posterior distributions for between‐protocol differences along with the central tendency (i.e., point estimate = mean) and highest density credible intervals. Negative values in Figure B favour greater (and more positive) FS ratings for RIR versus FAIL.

Raw FS ratings in weeks one, four, and eight are displayed in Figure 2 and Supplementary Table 3.3 and Bayesian estimates in Figure 5A. When averaged across each timepoint measured, greater (and more positive) FS ratings were estimated for RIR [1.2 (HDI: 0.7–1.8); *pd* = 100%] versus FAIL [0.3 (HDI: −0.3–0.8); *pd* = 86%]. Slightly greater FS ratings were also estimated for RIR [1.5 (HDI: 0.8–2.2); *pd* = 100%] versus FAIL [0.7 (HDI: 0.0–1.4); *pd* = 98%] in week 1, for RIR [0.8 (HDI: 0.0–1.7); *pd* = 97%] versus FAIL [0.2 (HDI: −0.6–1.0); *pd* = 68%] in week 4, and for RIR [1.4 (HDI: 0.6–2.3); *pd* = 100%] versus FAIL [–0.0 (HDI: −0.8 to 0.7); *pd* = 48%] in week 8. Estimates for between‐protocol differences are shown in Table 2 and posterior distributions in Figure 5B.

## 5 Discussion

### 5.1 Rating of Perceived Discomfort

We observed a 100% probability of slightly greater perceived discomfort following sets performed to momentary muscular failure versus 1‐ to 2‐RIR, with a difference of 0.9 (HDI: 0.6–1.2) points on the RPD scale when averaged over exercises and time points. These findings align with our previous acute experimental trial where RPD increased by ∼1 point from 3‐RIR to 1‐RIR, and from 1‐RIR to FAIL (M. C. Refalo et al. 2023a). Moreover, the largest difference in RPD between FAIL and RIR occurred in week eight [1.2 (HDI: 0.7–1.7) points], albeit only 0.4 points higher than weeks one and four. This was due to RIR experiencing a slight decrease in RPD from weeks one to eight (−0.5 points), whereas RPD for FAIL was very similar between weeks one and eight (−0.1 points). Differences in perceived discomfort between FAIL and RIR were also similar on the leg press and leg extension exercises (FAIL > RIR), with comparable average RPD values for both exercises. It is also possible that perceived discomfort would be greater if relative load was lower (and thus the repetitions per set higher) (Fisher et al. 2017); however, we chose the 8–10 and 10–12 repetition ranges to ensure perceptions of discomfort did not negatively influence RIR accuracy (Armes et al. 2020).

Previous research in recreationally trained individuals found an average RPD of 6.5 and 8.7 points when performing RT to momentary muscular failure with high and low loads, respectively (Fisher et al. 2017), which is substantially higher than the average RPD of FAIL in the present study (5.1 points). It is therefore possible that untrained individuals exhibit higher levels of perceived discomfort upon commencing RT that decreases with repeated exposure. Indeed, individuals may sometimes conflate perceived discomfort with exertion, despite these being distinct and independent constructs (Halperin et al. 2020). Although both perceptions tend to increase during exercise, they do not always correlate directly. For instance, prolonged efforts at lower intensities can elicit higher ratings of perceived discomfort compared to exertion (Halperin et al. 2020). It is worth noting that resistance‐trained individuals may demonstrate a greater ability to differentiate between perceived discomfort and exertion, potentially resulting in lower perceived discomfort ratings compared to untrained individuals. Our participants had ∼7.6 years of RT experience, which may explain the lower perceived discomfort ratings versus previous research and the minimal changes observed across the intervention. The higher perceived discomfort ratings observed for FAIL versus RIR are likely due to differences in neuromuscular fatigue between protocols, which is higher when sets are performed closer to momentary muscular failure (M. C. Refalo et al. 2024). For example, increased metabolite accumulation from additional repetitions performed (González‐Hernández et al. 2021) is a likely contributor to greater perceptions of discomfort when reaching momentary muscular failure. As such, individual differences in the rate of metabolite accumulation during an RT set, coupled with differences in pain tolerance, may contribute to the variability in perceived discomfort ratings observed across individuals performing the same task, as was seen in this study (Figure 2). Overall, resistance‐trained individuals experience slightly greater perceived discomfort when RT is performed to momentary muscular failure versus 1‐ to 2‐RIR. Little (RIR) to no (FAIL) reduction in RPD was observed from week one to eight, suggesting perceptions of discomfort remain relatively stable when the same RT protocol is repeated over time in resistance‐trained individuals. Overall, when considering our data and previous research (M. C. Refalo et al. 2023a), a decrease in RIR by ∼2 repetitions increases RPD by ∼1 point in resistance‐trained individuals lifting 8–12‐RM loads.

### 5.2 Rating of Perceived Exertion

Perceived exertion was greater when sets were terminated at momentary muscular failure versus 1‐ to 2‐RIR, with a difference of 1.1 (HDI: 0.6–1.6) points on the session‐RPE scale when averaged over all measured (*pd* = 100%). This difference was slightly smaller than the results of our previous acute experimental trial that found session‐RPE increased by ∼2 points from 3‐RIR to 1‐RIR, and from 1‐RIR to FAIL (M. C. Refalo et al. 2023a). Moreover, the largest difference in session‐RPE between FAIL and RIR occurred in week eight (1.3 points), albeit only 0.3 and 0.4 points higher than weeks one and four, respectively. This was due to RIR experiencing a slight decrease in session‐RPE from weeks one to eight (−0.5 points), whereas session‐RPE for FAIL was very similar between weeks one and eight (−0.2 points). Although average session‐RPE was 5.4 (HDI: 4.6–6.3) and 4.3 (HDI: 3.5–5.1) for FAIL and RIR, respectively, it is possible that perceived exertion could be even higher in an RT session involving more than two exercises. Although we attempted to design an ecologically valid RT protocol for the quadriceps, in practice individuals often perform exercises targeting other lower‐body muscles including the hamstrings, glutes, and calves within the same session. Further, various factors may influence ratings of perceived exertion with resistance exercise, such as the type of exercise undertaken (e.g., upper vs lower body), the load lifted and number of repetitions performed (Halperin et al. 2020).

We implemented the CR‐10 session‐RPE scale to represent the perceived exertion of a whole RT protocol and not that of the individual sets. In contrast, previous research has commonly used the ‘RIR‐based RPE scale’ to assess the perceived exertion of each set performed (Helms et al. 2016). For example, Mangine et al. (2022) stated that *“RIR = 3 would indicate that the subject could have completed an additional 3 repetitions during the set without assistance, and this would correspond to RPE = 7.”* The session‐RPE ratings in the present study are thus not indicative of any RIR values, but rather the difficulty of the whole RT protocol, as evidenced by a mean rating of 5.4 in FAIL despite all sets being performed at theoretically maximal exertion levels. Considering this, session‐RPE ratings across the intervention may have been influenced by extraneous factors such as day‐to‐day motivation, mood and focus, unlike the RIR‐based RPE scale, which is theoretically determined solely by the number of RIR. Nonetheless, controlling weekly RT volume progression through session‐RPE assessments produces comparable muscle hypertrophy and strength development to predetermined progression method, even when workload is reduced after two consecutive weeks of excessively high session‐RPE values (> 9) (Gomes et al. 2022). Similar muscle hypertrophy has also been observed between RT performed with 1‐ to 2‐RIR versus to momentary muscular failure (M. C. Refalo et al. 2024), despite the differences in perceived exertion observed in the present study. Overall, the findings of the present study suggest that perceived exertion of a whole RT session is greater when resistance‐trained individuals perform RT to momentary muscular failure versus with 1‐ to 2‐RIR, with little (RIR) to no (FAIL) change in session‐RPE observed from repeated exposure. Practically, session‐RPE ratings provide valuable insight into the overall exertion levels of an individual following RT and may inform RT programme design and weekly progression.

### 5.3 General Feelings

There were more positive general feelings following RIR versus FAIL, with an FS difference of 1.0 (HDI: 1.5 to 0.4) when averaged over all time points measured (*pd* = 100%). Our previous acute trial also found that FS decreased by ∼1 point from 3‐RIR to 1‐RIR, and from 1‐RIR to FAIL (M. C. Refalo et al. 2023a). Presently, we found that FS was 0.7 points lower for FAIL in week eight versus week one, suggesting repeatedly performing RT to momentary muscular failure may worsen post‐exercise feelings. Indeed, the largest difference in FS between protocols was 1.5 points in week eight (0.7 and 0.9 points higher than weeks one and four, respectively), but compatible with a range of 2.4 to 0.6 points, highlighting possible substantial differences favouring more positive feelings in RIR versus FAIL. Contributing to the greater differences in FS between FAIL and RIR observed in week eight was the more positive general feelings experienced by RIR from week four to week eight (Figure 5A). Considering that how one feels during and following exercise may influence long‐term adherence (Rhodes et al. 2015; Bastos et al. 2022), these differences may be practically important. Moreover, there is large inter‐individual variability in feelings towards a given RT protocol due to individual differences in *affective valence* (i.e., how much pleasure or displeasure an individual is likely to experience from exercise (Ekkekakis et al. 2011)); for example, individual FS scores ranged from −3 (‘fairly bad’) to +3 (‘good’) following RT to FAIL in week eight. Taken as a whole, performing RT with 1‐ to 2‐RIR promotes more positive general feelings compared to momentary muscular failure in resistance‐trained individuals. We also provide evidence that FS scores may worsen over time when RT is performed to momentary muscular failure but varies widely between individuals, such that some individuals experience positive feelings post‐exercise, whereas others, negative.

### 5.4 Strengths and Limitations of Current Research

Due to the scarcity of research, investigating perceptual responses to RT and their long‐term influence on adherence, it is unclear what magnitude of difference between protocols is practically relevant and how it would influence longer‐term outcomes. We therefore considered differences between protocols of 0.5–0.9 points as ‘slight’ (if *pd* = 100%), and differences ≥ 1 point as ‘meaningful’. We believe this is a reasonable classification, as our RT intervention consisted of only two exercises, and in practice individuals often perform more exercises, likely experiencing higher levels of discomfort, exertion and worsened feelings. Considering we chose to treat the ordinal outcomes as continuous within our statistical analysis, it is acknowledged that this approach may not fully capture the ordinal nature of the data. Nonetheless, to increase the precision of the outcome estimates, we included ‘participant sex’ and the ‘number of sets performed’ as population‐level effects in the statistical models, as we recruited both males and females and the number of sets performed increased by 20% halfway through the RT intervention. Although our sample consisted of 12 males and six females, with two limbs per participant we analysed 36 observations. Nonetheless, there were 50% more males than females, so we made no between‐sex comparisons. To limit the influence of our unilateral design on the outcomes, we (i) altered the starting limb each session to provide each limb an equal number of starting opportunities, (ii) only measured perceptual responses on the starting limb and (iii) assigned an equal number of dominant limbs to each RT protocol and accounted for correlations between limbs in our statistical models. To the best of our knowledge, this is the first RT intervention to compare perceptual responses across multiple timepoints. However, only three timepoints were assessed in the present study. Given the simplicity of such assessments, future research should aim to evaluate perceptual responses during and/or after each RT session to derive more valuable longitudinal insights.

### 5.5 Practical Application of Key Findings

An individual's perceptions of discomfort and exertion, along with their affective valence, should be considered when prescribing proximity‐to‐failure during RT. Importantly, each perceptual response provides unique insights into the individual experience of RT. For instance, RPD captures the physiological and unpleasant sensations associated with completing a specific task, session‐RPE reflects the perceived intensity of effort, strain or the amount of work required to complete an RT session, and FS conveys the overall emotional response or feelings associated with the completed RT session. Considering that (i) comparable muscle hypertrophy has been observed between RT performed to momentary muscular failure and 1‐ to 2‐RIR (M. C. Refalo et al. 2024) and (ii) maximum strength can be improved without reaching momentary muscular failure (Davies et al. 2016), individuals who experience more displeasure from exercise may perform a majority of their RT volume with 1‐ to 2‐RIR. Individuals may choose to reach momentary muscular failure more frequently if neutral or positive feelings are experienced. However, perceptual responses should be monitored over time to help inform periods of ‘deloading’ (i.e., period of intentionally and systematically reduced training demand (Bell et al. 2022)). For example, if for consecutive weeks during an RT intervention (i) feelings of sustained displeasure arise substantially, (ii) a given session‐RPE increases substantially despite no programme changes or (iii) the RPD of a given exercise increases substantially despite no changes to the proximity‐to‐failure reached, a period of deloading may be considered. Moreover, performing RT with 1‐ to 2‐RIR may ensure that perceived exertion and discomfort do not reach maximal levels, possibly allowing for improved adherence and more consistent and repeated bouts of RT that stimulate muscle hypertrophy and strength development. Assessing perceptual responses requires no costly equipment, major time investment or sophisticated analysis, and is a practical and effective strategy to retrieve instant feedback about how an individual feels during and/or following RT. We therefore recommend practitioners consider utilising RPD, session‐RPE and FS scales to monitor perceptual response to RT overtime and to ultimately promote desired outcomes whilst limiting negative feelings that may compromise long‐term adherence.

## 6 Conclusion

We report a 100% probability of (i) slightly greater perceived discomfort, (ii) greater perceived exertion and (iii) worsened general feelings, when performing RT to momentary muscular failure versus with 1‐ to 2‐RIR in resistance‐trained individuals. Perceptions of discomfort and exertion remained roughly similar across weeks but improved slightly for RIR. The largest differences in perceived discomfort and exertion between FAIL and RIR occurred in week eight (albeit only slightly greater than weeks one and four). Moreover, general feelings worsened over time for FAIL but varied widely between individuals, such that some individuals experienced positive feelings post‐exercise, whereas others, negative. Individuals who experience displeasure from performing RT to momentary muscular failure may benefit from terminating sets with 1‐ to 2‐RIR, potentially yielding comparable muscle hypertrophy and strength development (M. C. Refalo et al. 2024; Davies et al. 2016). Overall, assessing perceptual responses is a practical and effective strategy to retrieve instant feedback about how an individual feels during and/or following RT, which practitioners should consider utilising to promote desired outcomes whilst limiting negative feelings that may compromise long‐term adherence.

## Author Contributions

Article conceptualisation: M.C.R., J.J.F., E.R.H., D.L.H.; data collection: M.C.R.; drafted manuscript: M.C.R. and J.J.F.; statistical analysis: M.C.R.; critically revised manuscript: all authors. All authors read and approved the final manuscript.

## Ethics Statement

The study procedures were approved by the Deakin University Human Research Ethics Committee (reference number: 2022‐329). All participants read and signed a plain language statement.

## Consent

All participants provided consent for their data to be published.

## Conflicts of Interest

The authors declare no conflicts of interest.

## Supporting information

Supplementary Material

## Data Availability

All data and code utilised will be openly available on Open Science Framework: https://osf.io/34d92/.
