# Supplementary material for: The Effect of Proximity‐To‐Failure on Perceptual Responses to Resistance Training
Source: Eur J Sport Sci. 2025 Feb 17;25(3):e12266. doi: 10.1002/ejsc.12266 (PMC11832030; doi:10.1002/ejsc.12266)
Supplement: Supplementary file 1 — Supplementary Material [file EJSC-25-e12266-s001.docx]

# 1. Additional Content

## 1.1 Exercise Control During Resistance Training Intervention

To minimise any potential confounding influences on outcome measures, participants were allowed to perform additional moderate-intensity RT involving muscle groups other than the quadriceps (i.e., the hamstrings, gluteals, calves, and upper-body muscles) for a maximum of 20 sets per muscle group per week, however, additional RT of the hamstrings and gluteals was limited to specific exercises to reduce quadriceps engagement.

| **Exercise Exclusions and Inclusions** |
| --- |
| **Exercise Exclusions** |
| All quadriceps exercises, including variations of:   - Squat - Split squat - Lunge - Step up |
| All deadlift variations, including:   - Conventional deadlift - Sumo deadlift - Stiff leg deadlift |
| Barbell hip thrust (full range-of-motion) |
| **Exercise Inclusions** |
| Romanian deadlift (without full knee extension) |
| Hamstring curl machines |
| Cable pull-through (without full knee extension) |
| Hip abduction |
| Glute cable kickback (straight leg) |
| Glute bridge |
| Barbell or machine hip thrust (short range-of-motion) |
| Back extension |
| Glute-ham raise |

## 1.2 Visual Demonstration of Exercise Technique

*Range-of-motion was individualised for each participant using the safety mechanism on the leg press machine and lifting velocity of repetitions on the leg press were tracked with a GymAware device as shown in Figure A. Yellow dotted lines in Figure B show an example of how range-of-motion was assessed on the leg extension using an external implement with upper and lower range-of-motion limits that were individualised for each participant.*

# 2. Bayesian Data Analysis

## 2.1 Population-Level Effects and Group-Level Slope Structures (All Outcome Measures)

To account for dependent observations in each model, group-level intercepts were included for each participant and each limb was nested within each participant. A maximal group-level slope structure (i.e., population-level effects as group-level slopes) was initially attempted, but then simplified until no errors were generated upon fitting the model (Barr et al., 2013; Oberauer, 2022).

|  | **Population-Level Effects** | | **Group-Level Slopes** |
| --- | --- | --- | --- |
| **Outcome** | **Effects** | **Interactions** |  |
| Perceived discomfort | - Protocol (FAIL or RIR) - Exercise (leg press or leg extension) - Time (Weeks 1, 4, or 8) - Participant sex (Male or Female) - Number of sets performed (continuous) | - Protocol x Time x Exercise | - Protocol x Time x Exercise \| id - 1 \| id: limb |
| Perceived exertion | - Protocol (FAIL or RIR) - Time (Weeks 1, 4, or 8) - Participant sex (Male or Female) - Number of sets performed (continuous) | - Protocol x Time | - Protocol x Time \| id - 1 \| id: limb |
| General feelings | - Protocol (FAIL or RIR) - Time (Weeks 1, 4, or 8) - Participant sex (Male or Female) - Number of sets performed (continuous) | - Protocol x Time | - Protocol x Time \| id - 1 \| id: limb |

# 3. Additional Tables

## 3.1 Rating of Perceived Discomfort (Raw Values)

| **Variable - Protocol** | **Mean (kg)** | ***SD*** |
| --- | --- | --- |
| **WEEK 1** | | |
| Leg Press - FAIL | 5.8 | 1.9 |
| Leg Press - RIR | 4.7 | 1.9 |
| Leg Extension - FAIL | 5.2 | 2.2 |
| Leg Extension - RIR | 4.7 | 2.1 |
| Overall - FAIL | 5.5 | 1.9 |
| Overall - RIR | 4.7 | 1.9 |
| **WEEK 4** | | |
| Leg Press - FAIL | 5.2 | 1.7 |
| Leg Press - RIR | 4.5 | 1.5 |
| Leg Extension - FAIL | 4.8 | 1.7 |
| Leg Extension - RIR | 4.1 | 1.7 |
| Overall - FAIL | 5.0 | 1.6 |
| Overall - RIR | 4.3 | 1.5 |
| **WEEK 8** | | |
| Leg Press - FAIL | 5.4 | 1.8 |
| Leg Press - RIR | 4.3 | 1.5 |
| Leg Extension - FAIL | 5.3 | 1.7 |
| Leg Extension - RIR | 4.1 | 1.7 |
| Overall - FAIL | 5.4 | 1.6 |
| Overall - RIR | 4.2 | 1.5 |

## 3.2 Rating of Perceived Exertion (Raw Values)

| **Protocol** | **Mean (kg)** | ***SD*** |
| --- | --- | --- |
| **WEEK 1** | | |
| FAIL | 5.7 | 2.0 |
| RIR | 4.6 | 1.7 |
| **WEEK 4** | | |
| FAIL | 5.4 | 1.8 |
| RIR | 4.5 | 1.7 |
| **WEEK 8** | | |
| FAIL | 5.6 | 1.7 |
| RIR | 4.4 | 1.5 |

## 3.3 General Feelings via Feeling Scale (Raw Values)

| **Protocol** | **Mean (kg)** | ***SD*** |
| --- | --- | --- |
| **WEEK 1** | | |
| FAIL | 0.5 | 1.7 |
| RIR | 1.3 | 1.7 |
| **WEEK 4** | | |
| FAIL | 0.1 | 1.5 |
| RIR | 0.8 | 1.1 |
| **WEEK 8** | | |
| FAIL | –0.4 | 1.4 |
| RIR | 0.9 | 1.4 |

## 3.4 Repetitions-in-Reserve Prediction Accuracy

|  | **Leg Press** | |  | **Leg Extension** | |
| --- | --- | --- | --- | --- | --- |
| **RIR Target** | **Left** | **Right** |  | **Left** | **Right** |
| 1-RIR | ^↑^0.44 ± 0.51 | ^↑^0.44 ± 0.78 |  | ^↑^0.56 ± 0.51 | ^↑^0.44 ± 0.70 |
| 3-RIR | ^↑^0.61 ± 0.70 | ^↓^0.94 ± 1.16 |  | ^↓^0.83 ± 0.99 | ^↓^0.89 ± 1.08 |

Summary of absolute repetitions-in-reserve prediction accuracy (raw values) for both exercises on each lower limb. Arrow symbols inform the raw repetitions-in-reserve accuracy and indicate whether the average prediction was an overestimation (up arrow = ↑) or underestimation (down arrow = ↓). Data shown are presented as mean ± *SD*.

**3.5 Descriptive Characteristics for Each Resistance Training Protocol**

|  | **Week 1** | | | |  | **Week 2** | | | |
| --- | --- | --- | --- | --- | --- | --- | --- | --- | --- |
|  | **FAIL** | | **RIR** | |  | **FAIL** | | **RIR** | |
| **Variable** | **LP** | **LE** | **LP** | **LE** |  | **LP** | **LE** | **LP** | **LE** |
| Total Reps | 46 ± 10 | 57 ± 10 | 42 ± 7 | 63 ± 10 |  | 47 ± 8 | 59 ± 11 | 46 ± 9 | 63 ± 10 |
| Reps (first set) | 11 ± 3 | 11 ± 2 | 9 ± 2 | 12 ± 2 |  | 11 ± 2 | 12 ± 2 | 10 ± 2 | 12 ± 2 |
| Reps (final set) | 8 ± 2 | 8 ± 1 | 8 ± 1 | 9 ± 2 |  | 8 ± 2 | 8 ± 1 | 8 ± 2 | 10 ± 1 |
| *% Decrease Reps* | *23.5%* | *32%* | *8.7%* | *23.5%* |  | *17.9%* | *32.4%* | *11.6%* | *19%* |
| Load Lifted (kg) | 101 ± 45 | 32 ± 10 | 98 ± 43 | 31 ± 8 |  | 105 ± 44 | 31 ± 8 | 102 ± 42 | 31 ± 7 |
| Volume Load (kg) | 546 ± 289 | 254 ± 71 | 527 ± 273 | 253 ± 55 |  | 574 ± 268 | 244 ± 48 | 555 ± 254 | 246 ± 46 |
|  | **Week 3** | | | |  | **Week 4** | | | |
|  | **FAIL** | | **RIR** | |  | **FAIL** | | **RIR** | |
| **Variable** | **LP** | **LE** | **LP** | **LE** |  | **LP** | **LE** | **LP** | **LE** |
| Total Reps | 49 ± 11 | 60 ± 10 | 44 ± 9 | 64 ± 10 |  | 46 ± 8 | 63 ± 12 | 43 ± 8 | 63 ± 10 |
| Reps (first set) | 11 ± 4 | 12 ± 2 | 10 ± 2 | 13 ± 1 |  | 10 ± 2 | 13 ± 2 | 9 ± 2 | 13 ± 1 |
| Reps (final set) | 8 ± 2 | 8 ± 1 | 8 ± 2 | 10 ± 1 |  | 8 ± 2 | 9 ± 1 | 8 ± 2 | 9 ± 1 |
| *% Decrease Reps* | *21.6%* | *29%* | *8.1%* | *22.3%* |  | *21.4%* | *28.5%* | *11%* | *25.4%* |
| Load Lifted (kg) | 110 ± 46 | 31 ± 8 | 107 ± 43 | 31 ± 7 |  | 110 ± 47 | 32 ± 8 | 112 ± 44 | 32 ± 7 |
| Volume Load (kg) | 599 ± 282 | 248 ± 47 | 582 ± 266 | 252 ± 46 |  | 592 ± 266 | 257 ± 52 | 582 ± 255 | 264 ± 55 |
|  | **Week 5** | | | |  | **Week 6** | | | |
|  | **FAIL** | | **RIR** | |  | **FAIL** | | **RIR** | |
| **Variable** | **LP** | **LE** | **LP** | **LE** |  | **LP** | **LE** | **LP** | **LE** |
| Total Reps | 52 ± 11 | 63 ± 15 | 49 ± 11 | 66 ± 14 |  | 49 ± 12 | 65 ± 11 | 48 ± 10 | 65 ± 11 |
| Reps (first set) | 9 ± 2 | 12 ± 3 | 9 ± 1 | 12 ± 2 |  | 9 ± 2 | 13 ± 2 | 8 ± 2 | 12 ± 1 |
| Reps (final set) | 8 ± 1 | 8 ± 1 | 8 ± 1 | 9 ± 2 |  | 7 ± 2 | 8 ± 1 | 7 ± 1 | 9 ± 2 |
| *% Decrease Reps* | *20.2%* | *28.9%* | *10.6%* | *21%* |  | *26.8%* | *31.5%* | *15.2%* | *20.1%* |
| Load Lifted (kg) | 104 ± 41 | 34 ± 8 | 104 ± 42 | 34 ± 8 |  | 114 ± 45 | 34 ± 8 | 112 ± 43 | 35 ± 8 |
| Volume Load (kg) | 669 ± 225 | 303 ± 110 | 668 ± 227 | 313 ± 111 |  | 709 ± 237 | 300 ± 76 | 701 ± 219 | 307 ± 78 |
|  | **Week 7** | | | |  | **Week 8** | | | |
|  | **FAIL** | | **RIR** | |  | **FAIL** | | **RIR** | |
| **Variable** | **LP** | **LE** | **LP** | **LE** |  | **LP** | **LE** | **LP** | **LE** |
| Total Reps | 51 ± 8 | 67 ± 9 | 48 ± 10 | 69 ± 13 |  | 48 ± 10 | 68 ± 13 | 44 ± 9 | 73 ± 12 |
| Reps (first set) | 10 ± 2 | 13 ± 2 | 9 ± 2 | 12 ± 2 |  | 9 ± 3 | 13 ± 2 | 8 ± 2 | 13 ± 2 |
| Reps (final set) | 7 ± 2 | 8 ± 1 | 7 ± 2 | 9 ± 3 |  | 7 ± 2 | 8 ± 2 | 7 ± 2 | 9 ± 3 |
| *% Decrease Reps* | *32%* | *33.7%* | *21.7%* | *23%* |  | *25.6%* | *35.4%* | *20.7%* | *24.3%* |
| Load Lifted (kg) | 121 ± 45 | 36 ± 8 | 124 ± 44 | 37 ± 8 |  | 121 ± 48 | 37 ± 8 | 122 ± 47 | 37 ± 8 |
| Volume Load (kg) | 740 ± 274 | 344 ± 82 | 776 ± 253 | 331 ± 68 |  | 768 ± 287 | 330 ± 76 | 770 ± 277 | 338 ± 74 |

Repetition values are rounded to the nearest whole number. Percentage decrease from the first to final set is calculated from instances where the load was not adjusted across sets (i.e., not calculated from repetition data shown in table). Data shown are calculated as the average result from both resistance training sessions completed in each week and are presented as mean ± *SD*. *kg, kilograms; reps, repetitions.*

# 4. References

Barr, D. J., Levy, R., Scheepers, C., & Tily, H. J. (2013). Random effects structure for confirmatory hypothesis testing: Keep it maximal. *J Mem Lang*, *68*(3). <https://doi.org/10.1016/j.jml.2012.11.001>

Oberauer, K. (2022). The Importance of Random Slopes in Mixed Models for Bayesian Hypothesis Testing. *Psychol Sci*, *33*(4), 648-665. <https://doi.org/10.1177/09567976211046884>
